# Replication-Deficient Zika Vector-Based Vaccine Provides Maternal and Fetal Protection in Mouse Model

**DOI:** 10.1128/spectrum.01137-22

**Published:** 2022-09-28

**Authors:** Gustavo Garcia, Nikhil Chakravarty, Angel Elma Abu, Arjit Vijey Jeyachandran, Kari-Ann Takano, Rebecca Brown, Kouki Morizono, Vaithilingaraja Arumugaswami

**Affiliations:** a Department of Molecular and Medical Pharmacology, University of California, Los Angeles, Los Angeles, California, USA; b Department of Epidemiology, University of California, Los Angeles, Los Angeles, California, USA; c Department of Chemistry and Biochemistry, University of California, Los Angeles, Los Angeles, California, USA; d Division of Hematology and Oncology, Department of Medicine, David Geffen School of Medicine, University of California, Los Angeles, Los Angeles, California, USA; e AIDS Institute, David Geffen School of Medicine, University of California, Los Angeles, Los Angeles, California, USA; f Departments of Microbiology, Immunology, and Molecular Genetics, David Geffen School of Medicine, University of California, Los Angeles, California, USA; g Eli and Edythe Broad Center of Regenerative Medicine and Stem Cell Research, University of California, Los Angeles, Los Angeles, California, USA; h California NanoSystems Institute, University of California, Los Angeles, Los Angeles, California, USA; Regional Centre for Biotechnology

**Keywords:** Zika virus, *Flaviviridae*, arbovirus, pandemic potential, Zika congenital syndrome, microcephaly, Zika vector, RNA replicon, RNA vaccine platform, Zika vaccine, flavivirus

## Abstract

Zika virus (ZIKV), a mosquito-borne human pathogen, causes dire congenital brain developmental abnormalities in children of infected mothers. The global health crisis precipitated by this virus has led to a concerted effort to develop effective therapies and prophylactic measures although, unfortunately, not very successfully. The error-prone nature of RNA viral genome replication tends to promote evolution of novel viral strains, which could cause epidemics and pandemics. As such, our objective was to develop a safe and effective replication-deficient ZIKV vector-based vaccine candidate. We approached this by generating a ZIKV vector containing only the nonstructural (NS) 5′-untranslated (UTR)-NS-3′ UTR sequences, with the structural proteins capsid (C), precursor membrane (prM), and envelope (E) (CprME) used as a packaging system. We efficiently packaged replication-deficient Zika vaccine particles in human producer cells and verified antigen expression *in vitro*. *In vivo* studies showed that, after inoculation in neonatal mice, the Zika vaccine candidate (ZVAX) was safe and did not produce any replication-competent revertant viruses. Immunization of adult, nonpregnant mice showed that ZVAX protected mice from lethal challenge by limiting viral replication. We then evaluated the safety and efficacy of ZVAX in pregnant mice, where it was shown to provide efficient maternal and fetal protection against Zika disease. Mass cytometry analysis showed that vaccinated pregnant animals had high levels of splenic CD8^+^ T cells and effector memory T cell responses with reduced proinflammatory cell responses, suggesting that endogenous expression of NS proteins by ZVAX induced cellular immunity against ZIKV NS proteins. We also investigated humoral immunity against ZIKV, which is potentially induced by viral proteins present in ZVAX virions. We found no significant difference in neutralizing antibody titer in vaccinated or unvaccinated challenged animals; therefore, it is likely that cellular immunity plays a major role in ZVAX-mediated protection against ZIKV infection. In conclusion, we demonstrated ZVAX as an effective inducer of protective immunity against ZIKV, which can be further evaluated for potential prophylactic application in humans.

**IMPORTANCE** This research is important as it strives to address the critical need for effective prophylactic measures against the outbreak of Zika virus (ZIKV) and outlines an important vaccine technology that could potentially be used to induce immune responses against other pandemic-potential viruses.

## INTRODUCTION

The outbreak of Zika virus (ZIKV) in 2015 to 2016 attracted global attention due to the dire teratogenic effects caused by this mosquito-borne RNA virus, including microcephaly and fetal mortality in infants born to infected mothers (1). As such, intense research efforts to develop effective therapies and prophylactic measures have pressed on, although to little avail (2). The development of safe vaccines against ZIKV promises to have a major impact on global health and reduce potential epidemics in the future.

Viral vector-based vaccine platforms use an antigen-encoding region from a pathogen of interest inserted into the genome of a different virus that has been altered to be replication deficient and nonpathogenic (3). Replication-deficient vaccine models have been studied for decades, with replication-defective herpes simplex virus 1 (HSV-1) strains showing robust stimulation of anti-HSV-1 immune responses in mice in 1992 (4). Herpesviruses, poxviruses, and adenoviruses have traditionally been used as vaccine vectors, although replication-deficient RNA vaccine vectors have been developed in polioviruses and Sendai virus backgrounds, all showing induction of a durable immune response (5–9). Flavivirus-based vectors are limited, with most research focusing on Kunjin virus (KUNV). However, studies utilizing KUNV, West Nile virus (WNV), yellow fever virus (YFV), dengue virus (DENV), and tick-borne encephalitis (TBE) virus as expression vectors show increased ability to deliver and express genes of interest after infection (10–15). KUNV replicon-based vaccines were also found to be effective in developing immune responses against human immunodeficiency virus 1 (HIV-1) and simian immunodeficiency virus (SIV) antigens in mice and were evaluated to confer protection against Ebola virus in nonhuman primates (16–18). Replication-defective YFV, WNV, and TBE viral vector vaccine platforms, created through deletions in the viral structural proteins (capsid [C], precursor membrane [prM], and envelope [E] proteins [CprME]), were shown to be effective against HIV-1, WNV, Japanese encephalitis virus, YFV, and TBE (19–22). Adenoviruses have commonly been used as vectors due to their ability to infect a wide array of cell types, their effective expression of larger transgenes, and their production of high-titer vector particles *in vitro*, among other reasons (23). In an important milestone, adenoviral vector-based vaccines were approved against severe acute respiratory syndrome coronavirus 2 (SARS-CoV-2). The utilization of adenovirus-based vaccines against SARS-CoV-2, such as the AD26.COV2.S and ChAdOx1-S vaccines, is useful in preventing disease but not as effective as their mRNA counterparts (24–26). The strength of vector-based vaccines can be due to their intrinsic efficiency of delivering, translating, and presenting epitopes to antigen-presenting cells after vaccination. Our interest is to determine the use of ZIKV as a viral vector due to its ability to self-amplify its RNA genome through the expression of an RNA replication complex (RNA replicon) in the cytoplasm (27, 28).

Animal models serve as a crucial tool to determine vaccine safety and efficacy. Mouse models utilized to investigate ZIKV pathogenesis show that adult type I interferon (IFN) receptor-deficient (*Ifnar1^−/−^*) mice exhibit neurological disease and congenital infection when pregnant (29–32). Because wild-type immunocompetent adult mice can mount a potent type I IFN-mediated innate immune response and rapidly neutralize ZIKV infection, the *Ifnar1^−/−^* mouse model has been widely used as an *in vivo* model for ZIKV pathogenesis studies (33). Systemic and localized infection can be induced in female *Ifnar1^−/−^* mice through subcutaneous, intravaginal, or intrarectal injection of ZIKV, leading to manifestation of congenital and/or neurological symptoms (31, 34, 35). ZIKV infection of pregnant *Ifnar1^−/−^* female mice showed infection of the trophoblasts of maternal and fetal placenta, with fetuses showing reduced crown-rump length and occipito-frontal diameter of the head, a parallel to microcephaly in human fetuses (31, 36, 37). These risks of potential fetal infection certainly underlie a prevailing concern regarding ZIKV as a useful candidate as a viral vector-based vaccine. In this study, we outline an approach to develop a safe ZIKV-based vaccine and evaluate its efficacy in conferring protection against ZIKV to both pregnant and nonpregnant mice and developing fetuses.

## RESULTS

### Generation of ZIKV replication-deficient vector.

**FIG 1:**
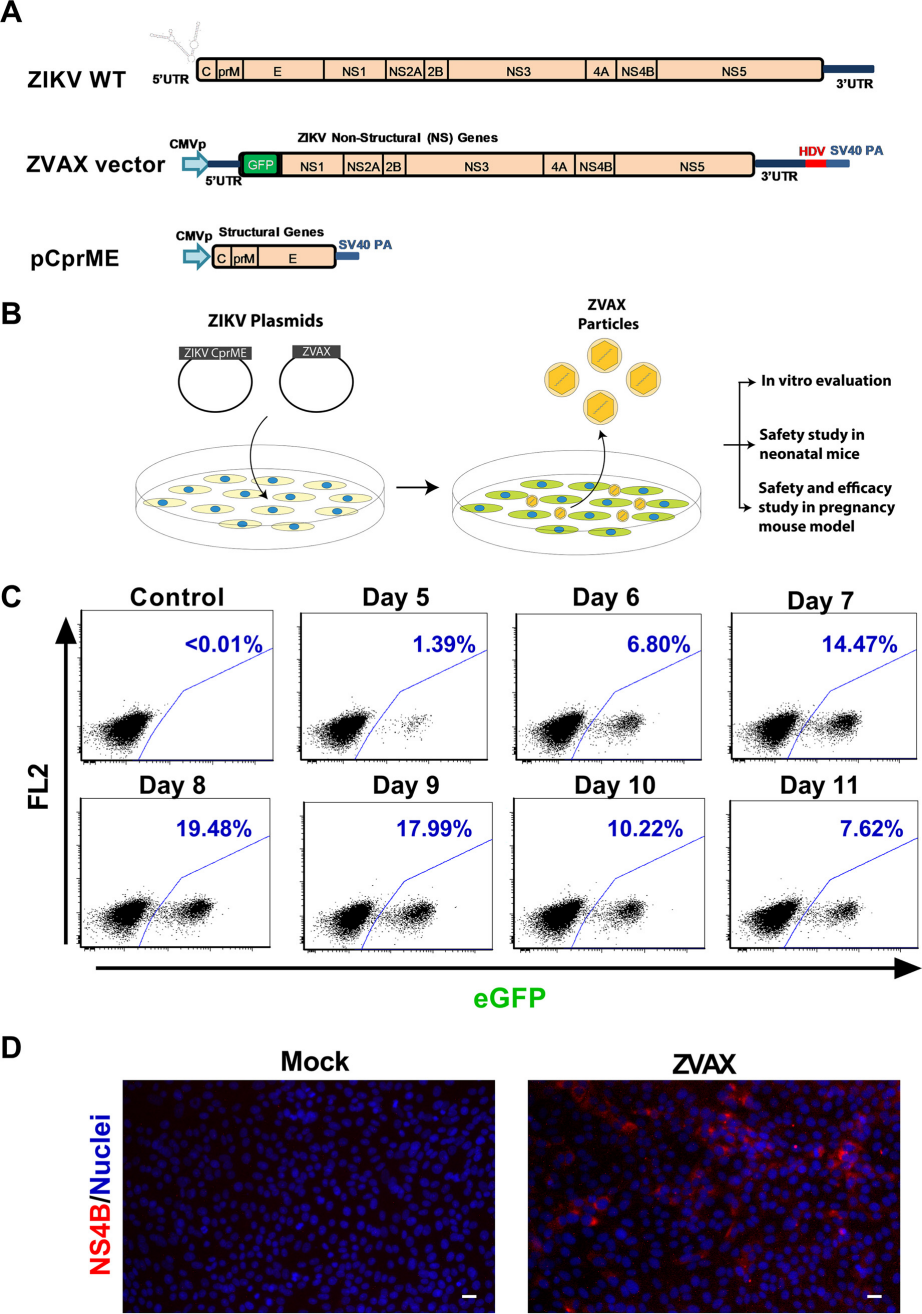
ZIKV vector constructs and vector packaging. (A) Genomic organization of wild-type Zika virus, replication-deficient Zika vaccine ZVAX vector, and packaging construct are presented. (B) Schematic illustration of the ZVAX packaging process and downstream analyses are depicted. (C) Flow cytometry histograms show the efficiency of ZVAX particle production by HEK 293T cells between days 5 and 11 posttransfection. The Vero cells were transduced with ZVAX vector particles produced from HEK 293T cells and analyzed for percentage of GFP-positive cells by flow cytometry at 2 days posttransduction. Untransduced Vero cells were used as negative controls. (D) Immunocytochemistry analysis of ZIKV NS4B antigen expression by ZVAX vector. Vero cells were inoculated with ZVAX vaccine particles and 48 h later were subjected to immunostaining analysis; red, NS4B; blue, nuclei. Scale bar, 25 μm.

We focused on generating a replication-deficient ZIKV that can enter the cells and express nonstructural (NS) viral proteins but not structural proteins. Endogenous expression of NS proteins is expected to present the peptides from those viral proteins on major histocompatibility complex (MHC) class I and induce CD8^+^ effector T cells. A similar replication-deficient WNV vector was previously generated by packaging a WNV replicon using ZIKV structural proteins in HEK 293T cells (38, 39). Cell-mediated immune response plays a critical role in controlling intracellular pathogens. We intended to induce cellular immunity against ZIKV NS proteins and examine its antiviral effects in animal models of ZIKV infection. Thus, we focused on generating a ZIKV replicon. We developed the replication-deficient vector system by splitting the ZIKV genome into structural and nonstructural regions (Fig. 1A). For the packaging vector, we utilized an expression plasmid (38) carrying the structural gene sequence from positions 108 to 2489 (2,382 nucleotides encoding 794 amino acids). To generate the vector construct, we deleted nucleotide positions 183 to 2405 of the PRVABC59 strain, resulting in the removal of 2,223 nucleotides encompassing the core (C), precursor membrane (PrM), and envelope (E) structural genes (ZIKV reference genome sequence KU501215.1). The Zika vaccine candidate (ZVAX) genomic insert is flanked by a cytomegalovirus (CMV) promoter at the 5′ end and a hepatitis delta virus (HDV) ribozyme/SV40 poly(A) sequence at the 3′ end (Fig. 1A). To quantify the titer of produced ZVAX particles, we engineered a green fluorescent protein (GFP) reporter gene downstream of the 5′ untranslated region (UTR) in place of a structural gene, which enabled us to measure the titer of vector particles using flow cytometry analysis of transduced cells. We first optimized conditions for vector particle production in HEK 293T cells. The cells were transfected with both the packaging plasmid (Zika structural genes [CprME]) and vector plasmid (ZVAX construct) at a ratio of 3:1 (Fig. 1B). Cell culture supernatant was collected daily from 5 to 11 days posttransfection (dpt). To confirm the titer and viral particle production efficiency, Vero cells (40, 41) were transduced with produced ZVAX particles at a 10-fold dilution. Flow cytometry analysis tracking expression of GFP in the cells at 48 h postransduction revealed that optimal production of vaccine particles occurred between days 7 and 9 posttransfection of plasmids in HEK 293T cells (Fig. 1C). Enhanced green fluorescent protein (EGFP) expression is mediated by NS proteins of ZVAX. Therefore, our flow cytometry data indicate endogenous expression of the ZIKV NS proteins in transduced cells. To confirm this, we directly examined expression of NS4B, one of the ZIKV NS proteins involved in RNA genome replication, by immunocytochemistry. We found that ZVAX-transduced Vero cells expressed the ZIKV NS4B intracellular antigen (Fig. 1D), which can contribute to the induction of cellular immunity against ZIKV. Taken together, we have established a ZIKV vector platform and optimum vector packaging conditions.

### The ZVAX candidate is safe in neonatal mice, and no revertant recombinant virus is generated.

**FIG 2:**
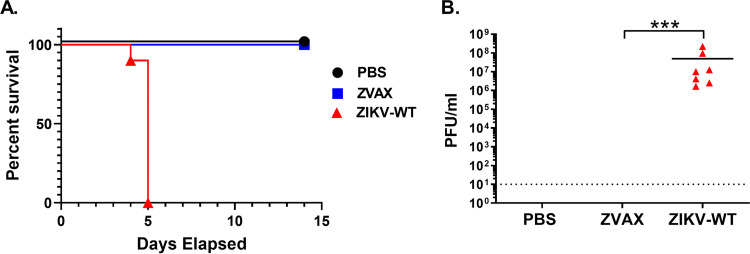
Safety evaluation of ZVAX in neonatal mice. (A) Kaplan-Meier survival graph shows percent mortality of inoculated pups (*n* = 7 per group). The wild-type ZIKV-infected pups exhibited 100% mortality. All the neonatal pups receiving ZVAX inoculum were viable. (B) ZIKV titer at 3 dpi. No replication-competent ZIKV was recovered from ZVAX-inoculated mice. A two-tailed unpaired, nonparametric Mann-Whitney test was performed; ***, *P* < 0.0001.

After producing vaccine particles, we evaluated the safety of the ZVAX vaccine candidate in neonatal *Ifnar1^−/−^* mice. Wild-type PRVABC59 ZIKV was included as a positive control. The pups were administered either the wild-type ZIKV virus (1 × 10^3^ PFU/mouse; intraperitoneal route) or ZVAX (1 × 10^3^ EGFP transduction units/mouse). After tracking survival over 14 days, we found that pups infected with wild-type virus had 100% mortality within 5 days postinfection (dpi), while those inoculated with ZVAX had no mortality and were all viable until the day 14 endpoint (Fig. 2A). After evaluating for animal viability, we determined viral load in both groups. Our data showed that, while wild-type ZIKV-infected pups showed a mean viral load of 10 million PFU/mL of blood, no infection was detected in the ZVAX-inoculated group at 3 dpi. This result indicates that the ZVAX vector was replication deficient, and no revertant virus was generated (Fig. 2B). These findings strongly suggest that ZVAX is a very safe vaccine and does not lead to ZIKV-mediated neonatal infection or mortality.

### The ZIKV vaccine candidate is effective in preventing ZIKV infection in adult nonpregnant mice.

**FIG 3:**
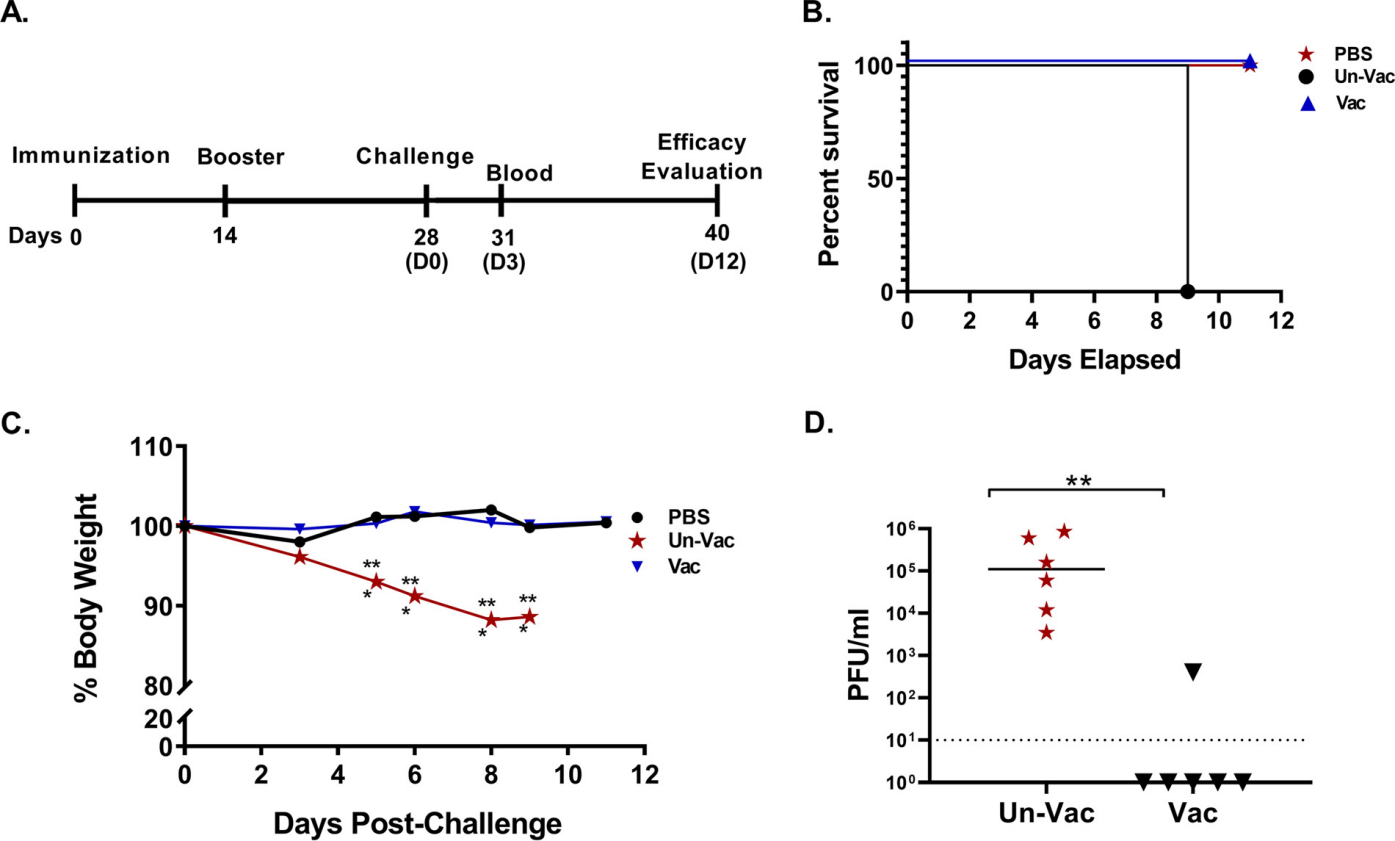
ZVAX preclinical safety and efficacy study in adult mice. (A) Schematic diagram of timeline. (B) Kaplan-Meier survival graph showing the mortality pattern of ZVAX-vaccinated (Vac) and unvaccinated (Un-Vac) adult mice (*n* = 7) after ZIKV lethal challenge. A mock-infected control group received PBS. (C) Body weight of wild-type PRVABC69 ZIKV-challenged vaccinated (ZVAX) and unvaccinated (Un-Vac) mice. Note that vaccinated animals have comparable body weight to the mock (PBS) challenged animals. (D) Serum viral load of unvaccinated and ZVAX immunized mice (*n* = 6) that were challenged with wild-type ZIKV (3 dpi). A two-tailed unpaired, nonparametric Mann-Whitney test was conducted; **, *P* < 0.001.

Adult nonpregnant *Ifnar1^−/−^* mice (mixed sex; 14 to 18 weeks old; *n* = 6 to 7 animals/group) were immunized with ZVAX (1 × 10^4^ EGFP transduction units/mouse; subcutaneous route), and the unvaccinated group received phosphate-buffered saline (PBS) injection. The mice were followed for 2 weeks. Body weight and animal mortality (if any) were the parameters monitored. All immunized mice survived and maintained normal body weight, thereby demonstrating that ZVAX is well tolerated. Because ZVAX is a replication-deficient subunit vaccine, one booster dose was given 2 weeks after primary immunization (1 × 10^4^ EGFP transduction units/mouse; subcutaneous route) to enhance the ZIKV-specific immune response. Then, at 4 weeks postprimary immunization, both the vaccinated and unvaccinated groups were challenged with wild-type ZIKV and were followed for 12 days. A schematic representation of this immunization timeline is presented in Fig. 3A. Mice vaccinated with ZVAX were protected from lethal ZIKV challenge (Fig. 3B) and body weight loss (Fig. 3C). Moreover, vaccination prevented viral replication at 3 days postchallenge (Fig. 3D). The unvaccinated mice developed significant weight loss, and 37% of the animals showed signs of posterior paralysis by 8 dpi. These results indicate that the ZVAX construct is safe and is effective in inducing a protective antiviral immune response against Zika disease.

### The ZIKV vaccine candidate provides protection against lethal ZIKV infection in pregnant mice.

**FIG 4:**
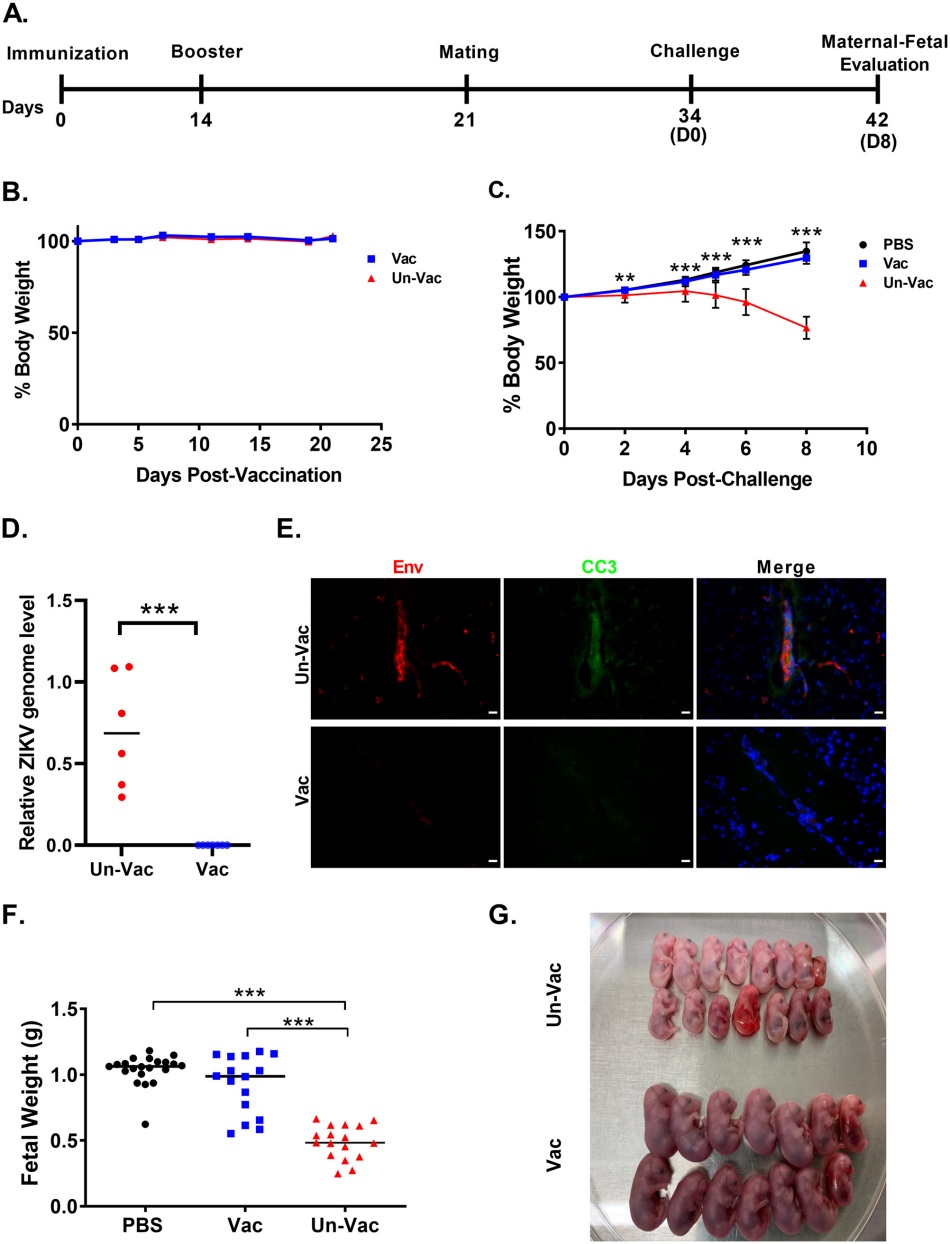
ZVAX immunization protects breeding females and fetuses from Zika viral disease. (A) Schematic diagram of immunization and key time points. (B) Percent body weight change in ZVAX-immunized (Vac; *n* = 10 mice) and unvaccinated (Un-Vac) groups (*n* = 9 mice). (C) Percent body weight change of ZIKV-challenged pregnant animals. Both vaccinated and unvaccinated groups were infected with PRVABC69 ZIKV. A mock-infected control group received PBS vehicle injection. (D) Relative ZIKV viral load in brains of unvaccinated and vaccinated pregnant animals at 8 days after ZIKV challenge. Brain viral RNA amount is shown as the ratio of ZIKV RNA copy numbers to GAPDH RNA copy numbers. A Student’s *t* test was performed; ***, *P* < 0.0001. (E) Representative images of IHC analysis of ZIKV-challenged pregnant animal brains depicts ZIKV-infected cells (red) and resulting apoptotic cell death (cleaved caspase-3 [CC3], green) in unvaccinated animals. Scale bar, 25 μm. (F) Body weight of fetuses of vaccinated and unvaccinated pregnant mice challenged with PRVABC69 ZIKV (8 dpi). The negative-control group received only PBS (mock infection). Multiple-comparison one-way analysis of variance (ANOVA) and Tukey tests were conducted; ***, *P* < 0.0001. (G) Growth retardation phenotype observed in fetuses from ZIKV-infected mothers.

After establishing a safety profile in neonatal mice and vaccine efficacy in adult nonpregnant mice, we evaluated the ability of the ZVAX candidate to immunize breeding female mice. The timeline of the key steps in the inoculation and process can be found in Fig. 4A. Female mice (*n* = 10) were immunized with ZVAX (1 × 10^4^ EGFP transduction units/mouse) through subcutaneous injection. Unvaccinated mice (*n* = 9) received a PBS vehicle. Vaccinated mice were then boosted at day 14. During the postimmunization period, vaccinated mice stayed healthy and active, suggesting tolerance and safety of ZVAX in adult mice. Body weight measurements in the vaccinated group were similar to those in unvaccinated mice throughout this phase (Fig. 4B). At day 21, both vaccinated and unvaccinated mice were subjected to timed mating. At 9 to 11 days postmating, the resultantly pregnant mice from both the vaccinated and unvaccinated groups were then challenged with wild-type PRVABC59 ZIKV (1 × 10^6^ PFU/mouse; subcutaneous route). A healthy mock-infected control pregnant mouse group was included, which received PBS injection. Vaccinated and mock-infected (PBS) pregnant mice gained weight, while unvaccinated pregnant mice lost weight and, by day 8 postchallenge, reached the euthanasia endpoint (Fig. 4C).

Subsequently, we analyzed mouse brain tissue for detecting virus replication and pathological apoptotic cell death. ZIKV viral load was measured by highly sensitive digital PCR, which revealed that ZVAX-immunized mouse brains had no detectable viral load, with higher viral load observed in unvaccinated challenged mice (Fig. 4D). Immunohistochemistry (IHC) analysis of pregnant unvaccinated mouse brains showed active ZIKV infection and apoptotic cell death, whereas vaccinated animal brains had no detectable viral antigen or apoptosis (Fig. 4E). Moreover, 100% of vaccinated mice were protected postchallenge. Importantly, we also observed that all fetuses of vaccinated mothers were healthy and maintained normal body weight (Fig. 4F). Fetuses from unvaccinated mothers had reduced body weight, and several were either reabsorbed or partially decomposed *in utero* (Fig. 4G). All pups born from the vaccinated group were healthy and similar to pups from the mock-infected group.

### ZVAX elicits a protective immune response.

We next attempted to investigate the mechanisms of protection against ZIKV infection by ZVAX. ZVAX endogenously expressed NS proteins in transduced cells; thus, we reasoned that presentation of NS protein peptides by MHC would induce cellular immunity against ZIKV NS proteins. Although the ZVAX transgene does not induce endogenous expression of ZIKV structural proteins, it is possible that the ZIKV E protein incorporated into the ZVAX vaccine particle may induce the generation of neutralizing antibodies against the ZIKV E protein.

**FIG 5:**
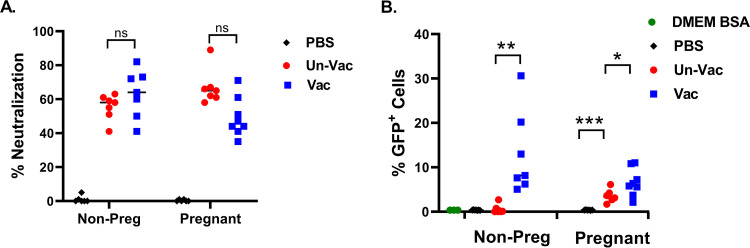
Quantifying levels of mouse serum neutralizing antibody and antibody-dependent enhancement of ZIKV infection. (A) Percent neutralization by mouse serum from either ZVAX-vaccinated challenged or unvaccinated challenged mice (8 dpi). No significant difference was observed between the vaccinated and unvaccinated groups. (B) Percent GFP-positive K562 cells to evaluate ADE response. Sera from vaccinated, challenged mice (8 dpi) exhibited a highly significant ADE response; 0.5% BSA and uninfected mice sera (PBS) samples were included as negative controls. A Student’s *t* test was performed; *, *P* < 0.01; **, *P* < 0.001; ***, *P* < 0.0001; ns, not significant.

To understand the cell-mediated and humoral immune responses induced by ZVAX, we set out to conduct antibody neutralization and antibody-dependent enhancement (ADE) assays as well as immunophenotyping of the cell-mediated immune response. We first analyzed neutralizing antibody response elicited by the vaccinated and unvaccinated ZIKV-challenged pregnant and nonpregnant mice through assessment of sera from each group with a highly sensitive neutralization assay using the ZIKV-EGFP replicon (Fig. 5A). We observed that the sera of ZIKV-challenged mice, regardless of ZVAX immunization status, had ZIKV-specific neutralizing antibodies (Fig. 5A). This suggests that mice exposed to ZIKV infection develop antibodies against the ZIKV structural proteins. Because the titers of anti-ZIKV neutralization antibodies do not differ between vaccinated and unvaccinated mice, it seems that T cell-mediated immunity, but not humoral immunity, plays a major role in the protection against ZIKV infection observed in ZVAX-vaccinated mice. Anti-ZIKV E protein antibodies were previously shown to induce ADE of ZIKV infection by bridging ZIKV E protein and Fc receptors on target cells (42). As such, we also performed an ADE assay using Fc receptor-positive K562 cells at the serum concentration that showed potent ZIKV-neutralizing activity. We found that the sera of vaccinated and challenged mice elicited significantly higher *in vitro* ADE than sera of unvaccinated and challenged mice (Fig. 5B). Given that the anti-ZIKV neutralizing antibody titers do not significantly differ between vaccinated and unvaccinated and challenged animals (Fig. 5A), these results suggest that the ZVAX virion-incorporated E protein induces a humoral response distinct from that induced by ZIKV infection alone.

**FIG 6:**
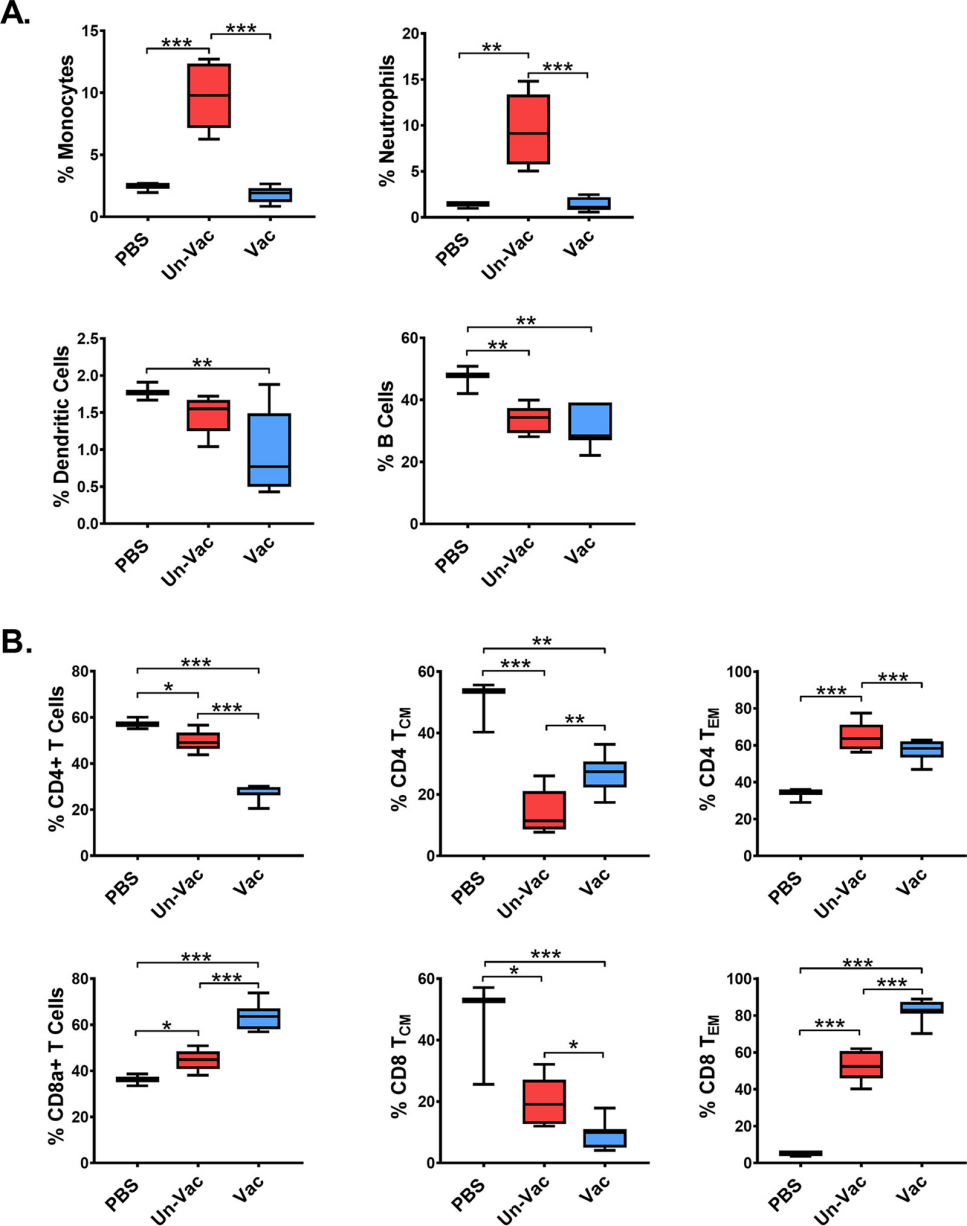
ZVAX immunization leads to protective immune responses against subcutaneous ZIKV challenge and T cell memory populations. (A) The percentages of splenic monocytes (CD45^+^CD11b^+^), neutrophils (CD45^+^Ly6G^+^CD11b^+^), conventional dendritic cells (cDCs; CD45^+^CD317^−^CD11c^+^), and B cells (CD45^+^CD3^−^CD19^+^) were determined for PBS and vaccinated and nonvaccinated mice at 8 days after subcutaneous ZIKV challenge. (B) Percentage of splenic CD4^+^ T cells, central memory CD4^+^ T cells (CD4^+^CD44^+^CD62L^+^), effector memory CD4^+^ T cells (CD4^+^CD44^+^CD62L^−^), CD8a^+^ T cells, central memory CD8^+^ T cells (CD8^+^CD44^+^CD62L^+^), and effector memory CD8^+^ T cells (CD8^+^CD44^+^CD62L^−^) at 8 days after subcutaneous ZIKV challenge. One-way ANOVA with Tukey’s multiple-comparison tests were conducted; *, *P* < 0.01; **, *P* < 0.001; ***, *P* < 0.0001.

ZIKV infection induced a drastic increase of the monocyte (identified using the CD11b marker) and neutrophil (identified using the Ly6 marker) populations in the spleen (33) compared to uninfected mice (Fig. 6A), indicating inflammatory responses after ZIKV infection. Consistent with the inhibitory effects against ZIKV replication and protective effects on the viability of infected mice, mice immunized with ZVAX showed a normal percentage of splenic monocytes and macrophages. These results indicated that ZVAX immunization enables a protective immune response without initiation of an inflammatory immune response after challenge with ZIKV. Interestingly, a significant decrease in dendritic cell and B cell expansion was observed in vaccinated mice compared to in mock-infected mice (Fig. 6A). Overall CD8a^+^ effector T cells were significantly higher in vaccinated mice than in unvaccinated mice (Fig. 6B). Previous studies found an increase in ZIKV-specific CD8^+^ effector T cell populations in *Ifnar1^−/−^* mice infected with wild-type ZIKV over the course of natural infection (43, 44). Moreover, memory T effector and central memory cell populations were significantly elevated in vaccinated populations compared to in unvaccinated mice, demonstrating activation of an adaptive T cell response. Therefore, the protection observed in the pregnant mice was likely the result of T cell-mediated immunity.

Together, these data suggest a potent antiviral immune response being activated in ZVAX-vaccinated mice without the deleterious inflammatory response. Altogether, these data show that our replication-deficient ZIKV vaccine is safe and effective in protecting pregnant mice.

## DISCUSSION

In this study, we developed a ZIKV vector-based vaccine that took advantage of a replication-deficient ZIKV construct to confer robust protection from lethal ZIKV infection without affecting fetal development through fetal infection or maternal disease. Flaviviruses provide an intriguing alternative to traditionally adenovirus-based vector vaccines due to their ability to amplify the expression of viral antigens through RNA replicons (41). ZIKV is known to infect the fetuses of an infected mother, resulting in microcephaly and other congenital brain abnormalities; thus, it is very important to establish rigorous safety data using a ZIKV vector vaccine (45). In the neonatal mouse model utilized in this study, we show that infection with our replication-deficient ZIKV vaccine construct did not cause mortality or neurological symptoms associated with Zika disease (Fig. 2 and 3). Given that viable pups were born to vaccinated mothers that were challenged with wild-type ZIKV and could maintain normal body weight and morphology, we can conclude a protective effect being conferred by ZVAX that does not induce any notable downstream adverse events.

We were also able to show that ZVAX-vaccinated adult mice challenged with wild-type ZIKV established a prominent adaptive immune response, resulting in blocked viral replication and no resultant inflammatory response. The immune profile observed in unvaccinated pregnant mice seems to be facilitated by proinflammatory mechanisms, as demonstrated by increased proliferation of monocytes and neutrophils, likely due to the upregulation of type I, type II, and type III IFNs as well as the activation of interferon-stimulated genes (ISGs) due to ZIKV infection (26, 46–49). Our findings about adaptive immune response are substantiated by other independent studies that found an increase in ZIKV-specific CD8^+^ effector T cell populations in *Ifnar1^−/−^* mice infected with wild-type ZIKV over the course of natural infection (43, 44, 50). Given that most studies have investigated primary infection with ZIKV, it stands to reason that titers of memory T cells would be low, as immunological memory would most likely not be well established over the course of the study. However, as we have observed in our results, with an increasing CD8^+^ T cell count increasing in ZVAX-inoculated mice, it can be inferred that there is a higher protective effect against ZIKV from the start of infection, resulting in a significant difference in mortality (or lack thereof) observed between the vaccinated and unvaccinated groups. Given the increase of effector memory T cells in the vaccinated group, vaccination with ZVAX may promote ZIKV-specific immunological memory. Compounded with the rise in CD8^+^ T cells, we can conclude that the significant increase in mouse viability after ZIKV challenge was largely promoted by ZVAX-mediated immunological memory and resultant rapid response of effector functions.

Our *in vitro* assay showed that vaccinated challenged mice generated similar titers of ZIKV-neutralizing antibodies as unvaccinated challenged mice. However, the sera of vaccinated, challenged mice induced significantly higher levels of ADE of ZIKV infection than the sera of unvaccinated challenged mice. Given the strong protective effects of ZVAX on *in vivo* viral replication and disease progression, it is unlikely that neutralizing antibodies play a significant role in the protective effect of ZVAX against ZIKV infection.

Most other studies conducted regarding ADE and ZIKV have examined the effect of cross-reactivity between antibodies for various flaviviruses, namely, dengue virus (DENV) and West Nile virus (WNV) (42, 51–57). In other *in vitro* studies, cross-reactive antibodies between ZIKV and DENV or WNV have been shown to elicit ADE, bringing concern of potential enhancement of Zika disease in those exposed to these other flaviviruses (51). However, *in vivo* studies to date have shown disparate results, with some suggesting that cross-reactivity between flavivirus antibodies may result in ADE (42, 52, 53), although there are several that showcase protective effects (42, 54–57). It is also worth noting that a presence of *in vitro* ADE response not observed *in vivo* is not unique to this study, with other independent studies examining vaccines against different types of viruses noting a similar phenomenon (58–60). Further experimentation is required in *in vivo* models to confirm the presence and effect of ADE using the ZVAX platform and to validate whether this is a concern to be noted and whether the cellular immunity elicited by the ZVAX platform would offset any deleterious outcomes from ADE, as we witnessed in both pregnant and nonpregnant mice. In addition, the cellular immunity and significant protection against ZIKV infection elicited by ZVAX with a lack of clear adverse events in vaccinated animals do suggest that this would be a safe vaccine.

However, this study is not without limitations. Given that we used an *Ifnar1^−/−^* animal model, which lacks full immunocompetence, these results are not totally representative of what may happen in immunocompetent animals as well as humans. Further studies need to be conducted in immunocompetent animals, particularly nonhuman primates, to further validate the efficacy of the ZVAX candidate. Moreover, given the clear benefits conferred by this novel ZIKV construct, further investigations ought to be conducted to see how ZIKV vector-based vaccines could induce immune responses against other pandemic-potential viruses, such as hantavirus, Ebola virus, and Chikungunya virus.

## MATERIALS AND METHODS

### Ethics statement.

This study was performed in strict accordance with the recommendations of the guide for the care and use of laboratory animals (61). All animal experiments were conducted under approved Institutional Animal Care Use Committee (IACUC) protocols (ARC-2017-068) at the University of California, Los Angeles.

### Viruses, cell lines, and plasmids.

The wild-type ZIKV strain used in this experiment was the PRVABC59 strain isolated in Puerto Rico in 2015 and obtained from the Centers for Disease Control and Prevention (CDC) (33). ZIKV stocks were prepared by infecting Aedes albopictus clone C6/36 (ATCC, CRL-1660) cells at 28°C and 5% CO_2_. The HEK 239T cell line was used for vaccine particle packaging. HEK 293T cells were cultured in Iscove’s modified Dulbecco’s medium (IMDM) containing 10% fetal bovine serum (FBS) and antibiotics at 37°C and 5% CO_2_. Vero E6 cells (ATCC) were cultured in Eagle’s minimum essential medium (EMEM; Corning) containing 10% FBS and antibiotics. K562 cells were cultured in IMDM containing 10% fetal calf serum (FCS) and 1% penicillin/streptomycin.

Plasmids expressing ZIKV structural genes (ZIKV H/PF CprME) were kindly provided by Benjamin Hurley, Theodore Pierson, and David Gordon (NIH) (38, 39). The ZVAX vector plasmid, pCMV-5′-UTR-NS-3′-UTR, was generated in this study by deleting the structural gene-coding sequence (nucleotide positions 183 to 2405 of the PRVABC59 ZIKV). A marker GFP gene-coding sequence was inserted at nucleotide position 183. CMV promoter/enhancer was precisely placed upstream of the 5′ UTR. Hepatitis delta virus (HDV) and SV40 polyadenylation signal sequences were cloned downstream of the 3′ UTR, thus allowing for correct generation of 3′-ZIKV subgenomic end following transcription. This vector design allows for ‘‘DNA-launched’’ replicon particle production.

### DNA-launched replicon vaccine production process.

HEK 293T cells were cultured in Iscove’s modified Dulbecco’s medium (IMDM) containing 10% fetal bovine serum (FBS) and antibiotics at 37°C and 5% CO_2_. One day before transfection, HEK 293T cells (1.4 × 10^7^ cells) were seeded in a T175 flask coated with 250 μg/mL rat collagen 1. Cells were then transfected with 37.5 μg of packaging plasmid (ZIKV H/PF CprME) and 12.5 μg of vector plasmid (ZVAX) using TransIT LT1 (Mirus Bio, Madison, WI) according to the manufacturer’s protocol. One day after transfection, cells were cultured at 30°C. Three days after transfection, culture medium was changed to serum-free AIM-V (Thermo Fisher Scientific) supplemented with antibiotics. Supernatant was collected every day from 5 to 11 days after transfection. After collection, cell matter and other debris were filtered out through filters (pore sizes of 0.22 or 0.45 μm) and centrifugation (2,000 × *g*, 10 min, 4°C). Filtered supernatant was subsequently frozen at −80°C. After harvesting the cell culture supernatant, the same volume was replaced by fresh medium. To measure vaccine particle production, Vero cells were inoculated with 250 μL of 1:10 diluted vaccine particles. Flow cytometry was performed 48 h after transduction.

### Preclinical safety study in neonatal mice.

Neonatal C57BL/6 background *Ifnar1^−/−^* mice (postnatal days 2 to 3) received 1 × 10^3^ PFU/mouse of PRVABC59 virus (positive control; *n* = 7), VAX-R1 and virus (*n* = 7), or PBS (*n* = 7) via intraperitoneal (i.p.) injection (20-μL volume) and were then monitored for 14 days. Mouse survival was tracked. For measuring virus titer, blood was collected at 3 dpi. Vero cells were used for quantitation of ZIKV. Cells were inoculated with 10-fold serially diluted serum, and at 48 to 72 hours postinfection (hpi), viral plaques were counted, and titers were calculated (27).

### Preclinical safety and efficacy study in adult mice.

Sexually mature 8- to 12-week-old breeding female *Ifnar1^−/−^* (strain B6.129S2-*Ifnar1^tm1Agt^*/Mmjax) mice were either immunized with ZVAX (*n* = 10 mice) or administered a vehicle of phosphate-buffered saline (PBS; *n* = 10 mice) via subcutaneous injection in the hindlimb region. Mice receiving the vaccine were boosted at day 14. For the nonpregnancy study, adult mice (vaccinated and unvaccinated) were challenged with wild-type PRVABC59 ZIKV (1 × 10^6^ PFU/mouse) via the subcutaneous route. At 3 dpi, blood was collected for virus titer measurement (*n* = 6). Mice were followed for 12 days to assess body weight changes and mortality. For the pregnancy study, both vaccinated (*n* = 10) and unvaccinated (*n* = 9) groups were subjected to mating on day 21 postimmunization. Pregnant mice were challenged with wild-type ZIKV (1 × 10^6^ PFU/mouse) on day 34 postimmunization (~9 to 11 days of pregnancy). Mock-infected animals (*n* = 8) received PBS injection. Mice were monitored daily for health checkup and body weight measurement. At 8 dpi, the animals were humanely euthanized for fetal and maternal tissue collection. Maternal and fetal body weights were measured, and changes in fetal growth and development were assessed.

### Digital PCR.

Mouse brain tissues were harvested in TRIzol (Invitrogen, USA), and total RNA was isolated as per the manufacturer’s recommendations. ZIKV genomes and glyceraldehyde-3-phosphate dehydrogenase (GAPDH) transcript were quantified by digital PCR using a QuantStudio Absolute Q and a Combinati SARS-CoV-2 wastewater surveillance assay kit (Thermo Fisher Scientific). The probes for ZIKV and mouse GAPDH were labeled by 6-carboxyfluorescein (FAM) and VIC, respectively, which enables quantitation of both genes from the same RNA samples. The sequences of the primers and probe for reverse transcription and droplet PCR were previously published (40). The RNA samples were diluted 400-fold with water. Two microliters of the diluted samples was mixed with Combinati 1-step reverse transcription master mix and the primers and probes for ZIKV and mouse GAPDH, followed by reverse transcription, digital PCR, and data analysis using QuantStudio Absolute Q. RNA levels were expressed as ZIKV genome copies per one copy of GAPDH in the mouse brain tissue.

### Antibody neutralization assay.

EGFP-expressing ZIKV replicon (hybrid with WNV) was produced as previously described (38, 39). Vero cells were seeded into 24-well plates 1 day before infection with ZIKV-EGFP replicon vector (2.5 × 10^4^ cells/0.5 mL of IMDM containing 10% FBS and 1% penicillin and streptomycin/well). ZIKV replicon was incubated in either the presence or absence of 0.5% mouse serum (1:100 dilution) for 30 min at room temperature, followed by inoculation of the cells at 37°C for 2 h. Subsequently, inoculum was replaced with fresh medium. The percentages of cells infected with the ZIKV replicon were analyzed by measuring EGFP expression by flow cytometry at 24 h postinfection. Percent neutralization was calculated with respect to uninfected control sera.

### Antibody-dependent enhancement assay.

ZIKV-EGFP replicon was incubated with 0.5% mouse serum (1:100 dilution) for 30 min at room temperature before infection. A negative control of DMEM containing 0.5% bovine serum albumin (BSA) was used for comparison in this assay. K562 cells were infected with the ZIKV replicon in the presence of mouse serum or BSA alone for 2 h at 37°C. The cells were cultured in 24-well plates with IMDM containing 10% FCS and 1% penicillin/streptomycin. Twenty-four hours later, infected cells were analyzed by measuring EGFP expression by flow cytometry.

### Mass cytometry.

Splenic immune cell populations were characterized through mass cytometry analysis (33). To characterize these populations, metal-conjugated antibodies were utilized for high-dimensional description of single-cell-level immune markers. We used the Maxpar cell surface staining protocol, abiding by the manufacturer’s recommendations (Fluidigm) with some minor modifications (33). Splenic cells were isolated from various groups at 8 days post challenge. The spleen was first incubated in cold 1× PBS, cut into pieces, and mechanically distributed using a syringe or 1-mL pipette tip. The resultant homogenates were filtered through sterile 70-μm cell strainers (Falcon cell strainer; Fisher Scientific) with 10 mL of cold 1× PBS. Red blood cells were lysed with ammonium chloride potassium (ACK) lysing buffer (Gibco) for no longer than 2 min at room temperature. Cells were resuspended in 8 mL of 1× PBS. About 3 × 10^6^ cells were aliquoted into a 1.5-mL screw-cap tube, centrifuged (300 × *g* for 5 min), and resuspended in 500 μL of 1× PBS with Cell-ID cisplatin (Fluidigm) for 5 min. Cells were subsequently resuspended in 50 μL of Maxpar staining buffer with Fc block solution (0.5 to 1 μg of CD16/CD32, clone 93; eBioscience) and incubated for 10 min. After incubation, we added 50 μL of metal-conjugated antibody cocktail with antibodies of interest (Table S1 in the supplemental material) at optimal concentrations and incubated for 30 min at room temperature. Cells were washed twice with Maxpar staining buffer before adding cell intercalation solution (Maxpar fix and perm buffer; Cell-ID Intercalator-Ir). Cells were then incubated overnight at 4°C. The next day (after overnight incubation), cells were washed with Maxpar staining buffer, resuspended in water, and subjected to mass cytometry. Individual cells were ionized and analyzed by a Helios CyTOF mass cytometer. Cell subpopulations were analyzed using CyTOF software v6.7 (Fluidigm) and FlowJo software (FlowJo, LLC). The absolute population of CD45^+^ cells was used for calculating the percentages of neutrophils, monocytes, dendritic cells, B cells, and T cells. CD4^+^ and CD8^+^ were total populations from CD3^+^ cells. Memory cells (central memory and effector memory) cells were relative populations within either CD3^+^CD8^+^ or CD3^+^CD4^+^ populations.

### Immunohistochemistry.

Cells or tissues were fixed in 4% paraformaldehyde (PFA) for 30 min or 1 h and washed three times with 1× PBS. Tissues were processed and sectioned as described previously (62). Cells and tissue sections were subsequently permeabilized and blocked using primary and secondary incubation buffer (1% bovine serum albumin [BSA], 0.3% Triton X-100, and 0.01% sodium azide in 1× PBS) for 1 h at room temperature. For ZIKV staining, cells were incubated overnight at 4°C with rabbit polyclonal anti-Zika NS4B antibody (1:250) or mouse monoclonal anti-envelope antibody (flavivirus group antibody [D1-4G2-4-15 {4G2}] [Genetex]). Apoptotic cells were detected using cleaved caspase-3 antibody (cleaved caspase-3 rabbit monoclonal antibody, clone D175 [Cell Signaling Technology, USA]). Cells were rinsed three times with wash buffer and incubated with fluorescently labeled secondary antibody for 1 to 2 h at room temperature. Subsequently, cells were rinsed three times with wash buffer for 5 min and stained with 4′,6-diamidino-2-phenylindole dihydrochloride (DAPI; Thermo Fisher Scientific) at a 1:5,000 dilution in 1× PBS for 10 min. Round coverslips with tissue sections were mounted on precleaned glass slides with Prolong gold antifade mountant (Thermo Fisher Scientific) and sealed using nail polish. Image acquisition was performed using Leica DMi1 fluorescence microscopes and the Leica Application Suite X (LAS X).

### Statistical analysis.

All testing was done with a two-sided alpha level of 0.05. Student’s *t* tests or nonparametric two-tailed unpaired Mann-Whitney *t* tests were used to determine statistical significance between the groups assessed (vaccinated versus unvaccinated) with GraphPad Prism software, version 8.3 (GraphPad Software, USA). Kaplan-Meier survival curves were analyzed using the Grehan-Kaplan-Wilcoxon test. *P* values of <0.05 were considered significant.
