# Supplementary material for: Replication-Deficient Zika Vector-Based Vaccine Provides Maternal and Fetal Protection in Mouse Model
Source: Microbiol Spectr. 2022 Sep 28;10(5):e01137-22. doi: 10.1128/spectrum.01137-22 (PMC9602260; doi:10.1128/spectrum.01137-22)
Supplement: Supplemental file 1 — . Download , file, 0.03 MB [file spectrum.01137-22-s0001.pdf]

Supplemental Table 1 (Related to Figure 6). Antibodies used for mass cytometry.

	Metal Conjugate (Isotopic label)	Target epitope	Antibody Clone	Source	Identifier
1	152Sm	CD3e	145-2C11	Fluidigm	3152004B
2	148Nd	CD11b (Mac-1)	M1/70	Fluidigm	3148003B
3	142Nd	CD11c	N418	Fluidigm	3142003B
4	89Y	CD45	30-F11	Fluidigm	3089005B
5	168Er	CD8a	53-6.7	Fluidigm	3168003B
6	145Nd	CD4	RM4-5	Fluidigm	3145002B
7	176Yb	CD45R (B220)	RA3-6B2	Fluidigm	3176002B
8	150Nd	Ly-6C	HK1.4	Fluidigm	3150010B
9	141Pr	Ly-6G	1A8	Fluidigm	3141008B
10	170Er	CD80 (B7-1)	16-10A1	Biolegend	104735
11	149Sm	CD19	6D5	Fluidigm	3149002B
12	147Sm	CD317	eBio927	Fisher (eBioscience)	50-145-15
13	139La	CD27	LG.3A10	Fluidigm	3150017B
14	160Gd	CD62L (L-selectin)	MEL-14	Fluidigm	3160008B
